# Genome-wide analysis of screen behaviors among adolescents identifies novel loci and overlap with educational attainment and mental disorders

**DOI:** 10.1038/s41598-025-17450-y

**Published:** 2025-10-02

**Authors:** Evgeniia Frei, Tahir Tekin Filiz, Oleksandr Frei, Robert Loughnan, Piotr Jaholkowski, Nadine Parker, Nora R. Bakken, Viktoria Birkenæs, Alexey A. Shadrin, Helga Ask, Ole A. Andreassen, Olav B. Smeland

**Affiliations:** 1 Centre for Precision Psychiatry, Oslo University Hospital HF, Division of Mental Health and Addiction, Psychosis Research Unit/TOP, Ullevål Hospital, Building 49, P.O. Box 4956, 0424 Nydalen, Oslo, Norway; 2 Division of Mental Health and Addiction, Oslo University Hospital, Oslo, Norway; 3 Centre for Bioinformatics, Department of Informatics, University of Oslo, Oslo, Norway; 4 Center for Population Neuroscience and Genetics, Laureate Institute for Brain Research, Tulsa, OK USA; 5 Center for Human Development, University of California, La Jolla, CA USA; 6 PsychGen Centre for Genetic Epidemiology and Mental Health, Norwegian Institute of Public Health, Oslo, Norway; 7 PROMENTA Research Center, University of Oslo, Oslo, Norway

**Keywords:** Behavioural genetics, Genetic association study, Genetics, Neurodevelopmental disorders

## Abstract

Technological devices play a central role in adolescents’ life. Despite concerns about negative effects of excessive screen time, there is little knowledge of screen behaviors’ genetic architecture. Using self-reports from adolescents in the Norwegian Mother, Father, and Child Cohort Study (*n* = 18,490), we performed genome-wide association analysis for four screen behaviors: time spent (1) watching television; (2) gaming; (3) sitting/lying down with a screen device; and (4) using social media. The resulting summary statistics were analysed using the conditional false discovery rate (condFDR) approach to increase genetic discovery. We also estimated SNP-heritabilities of the screen behaviors and genetic correlations with eight psychiatric disorders (schizophrenia, bipolar disorder, major depressive disorder, autism spectrum disorder, attention-deficit hyperactivity disorder, anorexia nervosa, cannabis use disorder and alcohol use disorder), and educational attainment. Screen behaviors displayed significant SNP-heritabilities (0.048–0.12). We observed significant genetic correlations between screen behaviors and psychiatric disorders (r_g_ range: 0.21–0.42). Educational attainment demonstrated negative genetic correlation with screen behaviors, most strongly with social media use (r_g_ = − 0.69). CondFDR analysis identified three novel loci associated with social media use. Thus, we show that screen behaviors are heritable, polygenic traits that partly share genetic signal with mental disorders and educational attainment.

**Supplementary Information:**

The online version contains supplementary material available at 10.1038/s41598-025-17450-y.

## Introduction

Technological devices have become an integral part of adolescents’ life. The majority of young people spend several hours per day on screen-based activities, and reports indicate that the numbers continue to increase^1^. Use of technology far outpaces our understanding of the fundamental features and health impact of screen behaviors, and additional research is needed. In the 1990s, the first evidence of genetic influence on television watching emerged, challenging the notion that it was a pure “environmental factor”^2^. Extensive research has since confirmed that screen behaviors are heritable traits^3^, including substantial twin heritability estimates of gaming behavior (19–63%)^4^, compulsive internet use (48%)^5^, and problematic internet use (58–66%)^6^ among adolescents. Recent work investigated SNP-based heritability (*h*^*2*^_*SNP*_) of screen behaviors in the Adolescent Brain Cognitive Development Study, and reported estimates that varied from zero to 10–18%, depending on the screen subtype^7^.

Despite this, the genetic architecture of screen behaviors among adolescents is poorly understood with a lack of studies detecting specific single nucleotide polymorphisms (SNPs)^8^. A recent genome-wide association study (GWAS) revealed several SNPs significantly associated with internet addiction disorder in adults^9^. Further, GWASs based on middle-aged individuals in the UK Biobank (UKB) cohort identified SNPs associated with television watching and leisure computer use^10^. However, it is unclear whether these results can be generalized to adolescents, who spend more time on screen devices than any other age group^11^.

Parallel to the widespread use of digital devices among young people, youth mental health problems are on the rise^12,13^. While excessive use of screen devices has been linked to negative mental health outcomes, the proposed explanations vary greatly^14–16^. Psychiatric disorders in children and adolescents are affected by genetic factors^17–19^, and the potential of shared genetic determinants underlying screen time and mental health problems in children and adolescents is a novel research topic^20^. Recent studies have indicated that genetic confounding may account for a substantial part of the phenotypic association between screen use and mental health^7,21^, and that major psychiatric disorders and screen behaviors may share a common genetic basis^20^.

Furthermore, a growing body of evidence suggests that increased screen time could affect academic performance in children and adolescents^22^. For example, gaming and social media use are associated with worse academic performance^23,24^. Evidence also suggests that prolonged screen time could contribute to a diminished capacity for sustained attention and a heightened susceptibility to distractions^25^. Finally, both twin studies and large GWASs have demonstrated that genetic factors are important for educational attainment^26,27^, although the extent to which academic performance and screen behaviors share genetic underpinnings remains unclear.

In this study, we leveraged data from the Norwegian Mother, Father, and Child Cohort Study (MoBa)^28^, a prospective population-based pregnancy cohort, to investigate the genetic architecture of screen behaviors among adolescents and their associations with key mental health traits and disorders. We aimed to identify specific genomic loci associated with screen behaviors in adolescents. To achieve this, we performed GWASs of four single screen behaviors (television watching, gaming, total screen time use, and social media use). We undertook extensive post-GWAS analyses, including estimating genetic correlations across screen behaviors and with eight major psychiatric disorders (schizophrenia [SCZ], bipolar disorder [BP], major depressive disorder [MDD], autism spectrum disorder [ASD], attention-deficit hyperactivity disorder [ADHD], anorexia nervosa [AN], alcohol use disorder [AUD], and cannabis use disorder [CUD]) – selected for their heritability, relevance to adolescence, and availability of large-scale GWAS data – as well as educational attainment (EA).

## Methods and materials

### Study sample

MoBa is a population-based pregnancy cohort study conducted by the Norwegian Institute of Public Health (NIPH)^28^. Participants were recruited from all over Norway from 1999 to 2008, and the women consented to participation in 41% of the pregnancies. The cohort includes approximately 114,500 children, 95,200 mothers and 75,200 fathers. Blood samples for genotyping were obtained from children (umbilical cord) at birth^29^. The current study is based on version 12 of the quality-assured data files released for research in January 2019, including all adolescents (14–16 years of age) with relevant phenotypic and genetic data available (*n* = 18,490).

The current study was approved by The Regional Committees for Medical and Health Research Ethics (2016/1226/REK sør-øst C), and all methods were carried out in accordance with relevant guidelines and regulations. All data and material in MoBa are collected with written informed consent from participants in the study. Children were included in the study after consent from the mother. The establishment of MoBa and initial data collection was based on a license from the Norwegian Data Protection Agency and approval from The Regional Committees for Medical and Health Research Ethics. The MoBa cohort is currently regulated by the Norwegian Health Registry Act.

### Screen behaviors

We used single item self-reports from the MoBa Q-14 year questionnaire (Supplementary Table 1). Specifically, adolescents reported how much time they spent on the following screen-based activities per the average weekday: (1) watching movies/series/TV; (2) gaming; (3) sitting/lying down with a screen device (irrespective of activity); (4) communicating with friends on social media.

### Genome-wide association analyses

GWASs were conducted using an additive multivariate linear regression model with PLINK2 on a sample of 16,027 unrelated individuals (see Supplementary Note for details)^30^. The first twenty genetic principal components (PCs), age, sex, and genotyping batch (N = 26, as factors) were used as covariates. The analyses were restricted to individuals of European ancestry.

### Conditional false discovery rate (condFDR) analyses

To improve statistical power and genetic discovery, we analyzed the resulting summary statistics using the condFDR approach^31,32^ (see Supplementary Note for more details). In our study, the primary phenotypes were the four screen time measures, with educational attainment as a secondary phenotype^27^. To facilitate evaluation of identified loci in the UK Biobank, we excluded this cohort from the EA summary statistics. The FDR significance cut-off was set at 0.01, in line with the previous literature^31,32^.

### Evaluation of the identified Loci in an Independent Sample

We used GWAS results from the UKB cohort on leisure television watching (TV-UKB) and leisure computer use (PC-UKB) to test whether our results can be supported by data from an independent sample^10^. For this purpose, we checked whether effects of the lead SNPs identified by condFDR analysis are consistent between the MoBa and UKB data sets. Additionally, we obtained the *p*-values of the lead SNPs from the MoBa cohort in the UKB sample.

### Functional analyses

The lead SNPs were mapped to putative causal genes using the Variant to Gene (V2G) tool from the open-source OpenTargets Genetics^34^. This platform was also used to inspect associations of the mapped genes with other phenotypes.

### Estimation of SNP-based heritabilities and genetic correlations

*h*^*2*^_*SNP*_ of screen behaviors were estimated from the GWASs summary statistics using linkage disequilibrium score regression (LDSC)^35^. To estimate *h*^*2*^_*SNP*_ using individual genotype data, we conducted GCTA-GREML analysis^36^.

We also applied bivariate LDSC^35^ to estimate genetic correlations (r_g_) across screen behaviors and with eight major psychiatric disorders (SCZ, BP, MDD, ADHD, ASD, AN, AUD, CUD)^37–44^, as well as EA^27^. The selected psychiatric phenotypes reflect a combination of prevalent conditions with available large-scale GWAS data, most of which typically emerge during adolescence or young adulthood. Genetic correlations were estimated in the main study sample, as well as in the subsample of participants without a history of a psychiatric disorder. We also estimated genetic correlations between the screen behaviors in MoBa and TV watching and leisure computer use in the UKB cohort.

Correlations are presented as the coefficient ± standard error. Original *p*-values are reported. Multiple testing correction was performed using the Benjamini–Hochberg procedure with FDR < 0.05.

### Sensitivity analysis

To ensure that the presence of psychiatric diagnoses in the study sample does not confound the results, we performed a sensitivity analysis and re-estimated all genetic correlations using GWAS on screen phenotypes based on subsample of participants without a history of any psychiatric disorder. Of the 18,490 participants with relevant phenotypic and genetic data available, 3705 (20.04%) had at least one psychiatric diagnosis (Supplementary Table 2). We also conducted the Mendelian Randomization (MR)^46^ analysis (see Supplementary Note for details).

Given the substantial genetic overlap between many psychiatric disorders and educational attainment (EA), we used genomic structural equation modelling (SEM)^45^ to assess how the genetic correlations between screen-based behaviours and psychiatric disorders change when adjusting for shared genetic influences with EA (see Supplementary Note for details).

We also conducted the Mendelian Randomization (MR) analysis^46^ (see Supplementary Note for details).

## Results

In total, 18,490 participants had relevant phenotypic and genetic data available, and 16,027 unrelated individuals were included in the genetic analysis. Basic demographic characteristics of the initial sample are presented in Table 1. Descriptive information on study variables is presented in the Supplementary Table 1.

**Table 1:** Basic demographic characteristics of the adolescent sample.

Demographic characteristics of the adolescent sample (*n* = 18 490) from MoBa	
Age when questionnaire was answered, years, mean (SD)	14.42 (0.51)
Sex assigned at birth, *n* (%)	
Male	8,621 (46.63%)
Female	9,58 (53.32%)
NA	11 (0.059%)
Gender identity, *n* (%)	
Boy	8,369 (45.26%)
Girl	9,527 (51.53%)
Trans person	42 (0.23%)
Do not know / Do not wish to answer	254 (1.37%)
NA	298 (1.61%)

NA indicate missing values.

### GWASs of screen behaviors

Detailed information about missing values (≤ 1.5% for any phenotype), and sample sizes for each screen-based phenotype are available in the Supplementary Table 1. There was no evidence of stratification artefacts or uncontrolled test statistic inflation (Supplementary Figs. 1–2). No SNP reached genome-wide significance (*p* < 5 × 10^−8^) in any of the four GWASs. Lists of SNPs that reached the suggestive genome-wide significance level (*p* < 1 × 10^−5^) are given in the Supplementary Tables 4–7.

### SNP-based heritability

**Fig. 1:**
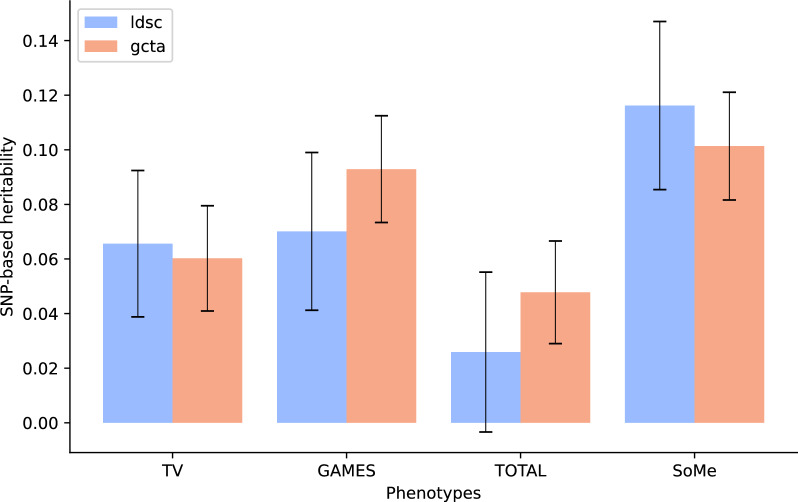
Single nucleotide polymorphism–based heritability (h^2^_SNP_) estimates for screen behaviors, obtained with LDSC (blue bars) and GCTA-GREML (orange bars). Error bars indicate standard errors of the estimated values. TV: watching movies/series/TV; GAMES: playing games on PC, TV, tablet, mobile, etc.; TOTAL: sitting/lying down with PC, mobile or tablet; SoMe: communicating with friends on social media.

LDSC results showed that *h*^*2*^_*SNP*_ for television watching, gaming, and social media use was 0.066, 0.070, and 0.12, respectively (Fig. 1, Supplementary Table 3). LDSC heritability estimate of the total screen time phenotype was not significantly different from zero.

GCTA-GREML produced concordant heritability estimates. Specifically, *h*^*2*^_*SNP*_ of television watching, gaming, and social media use was 0.060, 0.093, and 0.10, respectively. Total screen time use *h*^*2*^_*SNP*_ estimated with GCTA-GREML was 0.048.

### Identification of loci associated with social media use

Conditional QQ plots demonstrated enrichment of SNP-associations with social media use conditional on increasing levels of significance with EA (Supplementary Fig. 3).

**Fig. 2:**
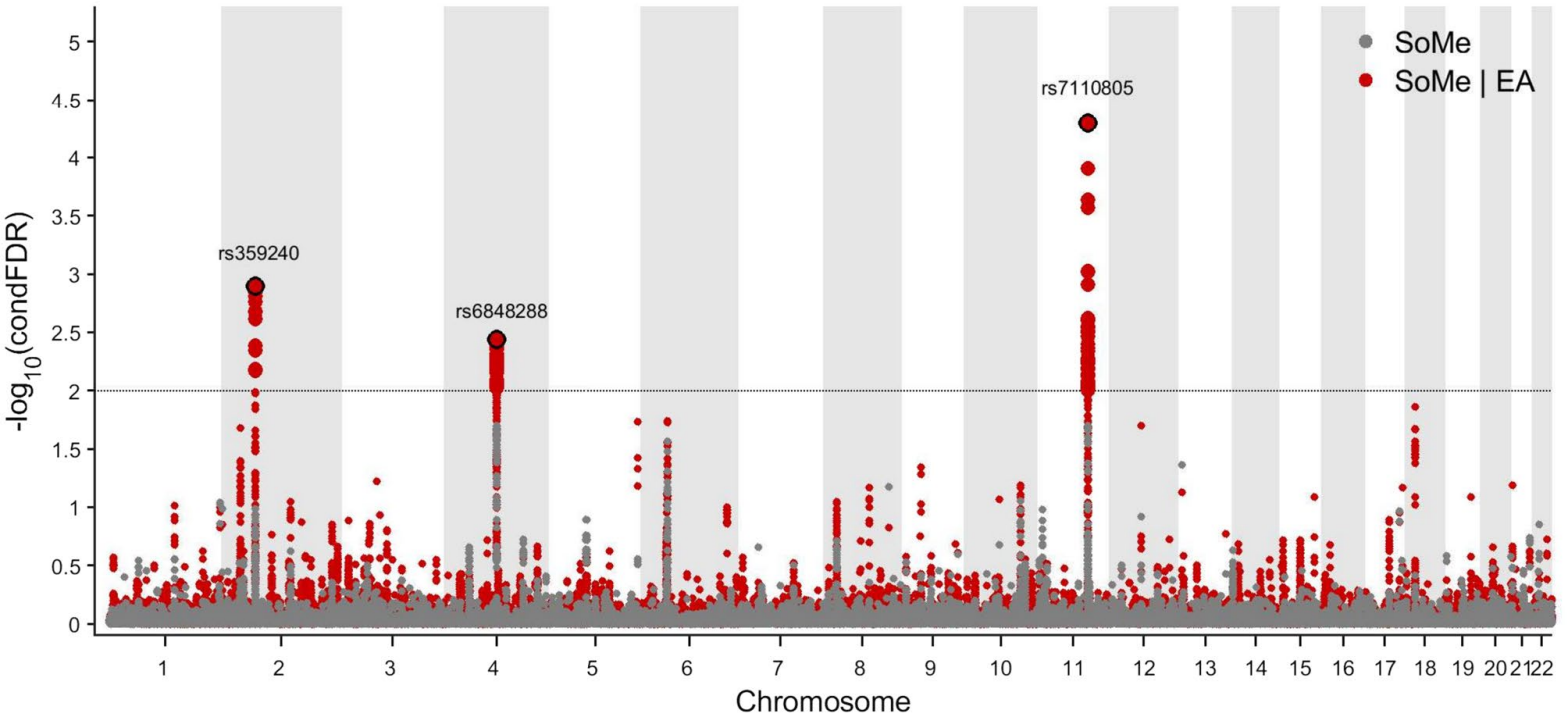
Common genetic variants significantly associated with social media use (SoMe) among adolescents in the MoBa sample. The variants were identified at conditional false discovery rate (condFDR) < 0.01 after conditioning on educational attainment (EA). The Manhattan plot displays the –log10 transformed condFDR values for each single-nucleotide polymorphism (SNP) on the y-axis with chromosomal positions along the *x*-axis. The small points represent non-significant SNPs, the bold points represent significant SNPs (condFDR < 0.01). Points corresponding to significant SNPs with lowest conditional FDR in each linkage disequilibrium (LD)-independent region (r^2^ > 0.10) have the rs-number written above it. The horizontal grey dotted line shows the significance threshold of condFDR (0.01). Gray dots stand for unconditional FDR values. SoMe: communicating with friends on social media.

**Fig. 3:**
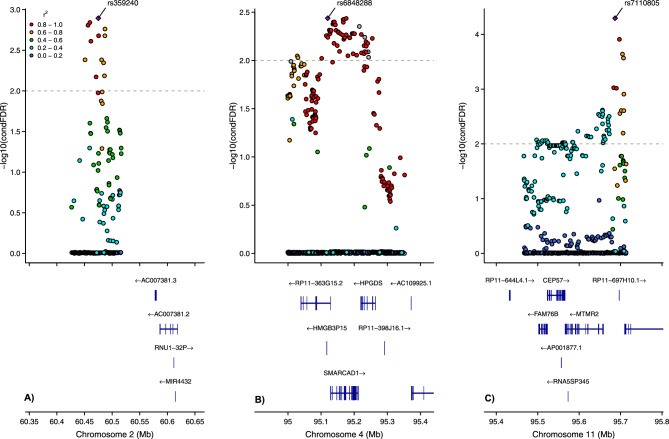
The genetic context of the strongest associations identified in the conditional false discovery rate (condFDR) analysis. Values for variants occupying the locus are shown on the left *y*-axis as –log10(condFDR). In each subplot, a single nucleotide polymorphism (SNP) with the strongest association is shown in the large purple square. The colour of the remaining markers reflects the degree of linkage disequilibrium (LD) with the strongest-associated SNP measured as r^2^ coefficient (described in the legend). The dotted line indicates the condFDR threshold of 0.01. (**A**) surrounding of rs359240 (condFDR = 1.28 × 10^−3^). (**B**) surrounding of rs6848288 (condFDR = 3.65 × 10^−3^). (**C**) surrounding of rs7110805 (condFDR = 5.10 × 10^−5^).

We leveraged this cross-trait enrichment using condFDR analyses and identified three LD-independent loci associated with social media use at condFDR < 0.01 (Fig. 2). The lead SNPs in the identified loci were mapped to putative causal genes using the V2G tool from the Open Targets Genetics^34,48,49^. The strongest signal was located at an intergenic variant (rs7110805, condFDR = 5.10 × 10^−5^), on chromosome 11 (Fig. 3C). Its nearest gene is *MTMR2*, while the region also contains the genes *FAM76B* and *CEP57* (downstream). Three additional independent significant SNPs (rs1727149, rs10765775, rs1893057) were identified in this large region spanning more than 250,000 bp. The second strongest independent condFDR signal was an intergenic variant on chromosome 2 (rs359240, condFDR = 1.28 × 10^−3^, Fig. 3A). No genes were residing in the direct vicinity of this SNP, and only 34 SNPs were in strong LD (r^2^ > 0.6). Nevertheless, rs359240 has a high CADD score of 19.6^50^. Finally, the condFDR analysis identified an ncRNC intronic variant on chromosome 4 (rs6848288, condFDR = 3.65 × 10^−3^, Fig. 3B), with nearest protein-coding gene *SMARCAD1*. rs6848288 tags a broad region of associations, covering around 270,000 bp, and has 139 candidate SNPs in strong LD (r^2^ > 0.6). Besides *SMARCAD1*, this region also contains the *HPGDS* gene (upstream)*.*

### Evaluation of the identified loci in an independent sample

We examined the identified loci in the association summary statistics from the independent TV-UKB and PC-UKB GWASs^10^. We also evaluated the respective genetic correlations. PC-UKB was significantly correlated with gaming, but not with the other MoBa phenotypes, whereas TV-UKB showed positive genetic correlations with three screen behaviors in MoBa (r_g_ = 0.38–0.52, see Supplementary Table 12). Positive genetic correlations warrant evaluation of identified loci in TV-UKB and PC-UKB, despite considerable differences between the MoBa and the UKB cohorts in age and phenotype definitions.

Locus 3, represented by rs7110805, has the same direction of effect in the MoBa (social media use) and TV-UKB samples, with *p* < 0.05. Locus 1 and locus 2, represented by rs359240 and rs6848288, respectively, have the same direction of effect in the MoBa (social media use) and PC-UKB samples. Moreover, the *p*-value for rs359240 in the PC-UKB sample was nominally significant (*p* < 0.05). These positive evaluation results reassure validity of the identified loci.

### Genetic overlap with key mental traits and disorders

**Fig. 4:**
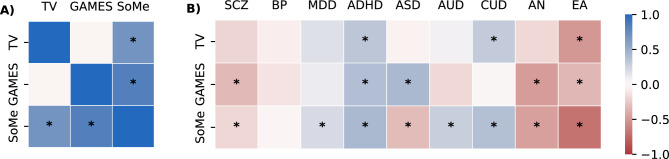
Genetic correlation estimates (**A**) among screen behaviors and (**B**) between screen behaviors and eight major psychiatric disorders and educational attainment. Asterisks indicate significant estimates at FDR < 0.05 (Benjamini–Hochberg procedure). TV: watching movies/series/TV; GAMES: playing games on PC, TV, tablet, mobile, etc.; SoMe: communicating with friends on social media; SCZ, schizophrenia; BP, bipolar disorder; MDD, major depressive disorder; ASD, Autism spectrum disorder; ADHD, Attention-deficit hyperactivity disorder; AUD, Alcohol use disorder; CUD, Cannabis use disorder; AN, Anorexia nervosa; EA, Educational attainment.

We evaluated pairwise genome-wide genetic correlations between the three screen-based phenotypes with significant LDSC estimated *h*^*2*^_*SNP*_ (TV watching, gaming, social media use) and six major psychiatric disorders, as well as EA. In addition, we estimated genetic correlations between the screen-based phenotypes themselves. The results are shown in Fig. 4, and in Supplementary Tables 9–11. We corrected for multiple comparisons using FDR < 0.05.

We identified significant genetic correlations between several screen time measures. Specifically, social media use was positively correlated with gaming (r_g_ = 0.83, SE = 0.27, *p* = 0.0021) and TV watching (r_g_ = 0.69, SE = 0.25, *p* = 0.0065), while TV and gaming were not significantly correlated.

We observed significant genetic correlations between screen behaviors and psychiatric disorders (r_g_ in range 0.21–0.42). ADHD showed moderate positive genetic correlations with TV watching (r_g_ = 0.33, SE = 0.12, *p* = 0.006), gaming (r_g_ = 0.39, SE = 0.13, *p* = 0.0036), and social media use (r_g_ = 0.42, SE = 0.09, *p* = 3.67 × 10^−6^). ASD was positively correlated with gaming, but negatively with social media use. Both MDD and AUD were positively correlated with social media use (r_g_ = 0.21, SE = 0.065, *p* = 0.0012, and r_g_ = 0.31, SE = 0.12, *p* = 0.020, respectively), while SCZ was negatively correlated with gaming (r_g_ = -0.30, SE = 0.12, *p* = 0.0004). CUD showed a modest positive genetic correlation with TV watching (r_g_ = 0.30, SE = 0.13, *p* = 0.019), and a stronger correlation with social media use (r_g_ = 0.38, SE = 0.09, *p* = 1.03 × 10^−5^). AN demonstrated substantial negative genetic correlation with both gaming (r_g_ = − 0.48, SE = 0.14, *p* = 0.0007) and social media use (r_g_ = − 0.46, SE = 0.10, *p* = 4.41 × 10^−6^). Finally, EA showed significant negative genetic correlation with all three screen behaviors, most strongly with social media use (r_g_ = -0.69, SE = 0.097, *p* = 9.38 × 10^−13^).

As a sensitivity analysis, we re-estimated all genetic correlations using the subsample of participants without a history of any psychiatric disorder. The estimates in the two samples were highly concordant (r = 0.987, *p* = 1.84 × 10^−21^; see Supplementary Table 10, Supplementary Fig. 4). Additionally, we re-calculated the genetic correlations between screen behaviors and psychiatric disorders while conditioning on EA. Several associations were attenuated; however, the residual correlations between social media use and anorexia nervosa, as well as between gaming and both autism spectrum disorder and anorexia nervosa, remained statistically significant after correction for multiple testing (see Supplementary Tables 14, 15).

In an attempt to address the question of causal relationships between screen behaviors and mental disorders, we applied MR analyses. No significant causal relationships were detected (see Supplementary Note, Supplementary Table 13), likely due to the limited statistical power of the analysis.

## Discussion

The present study investigated the genetic architecture of screen behaviors among adolescents and their associations with mental disorders and educational attainment (EA). Leveraging one of the largest birth cohorts in the world^28^, we demonstrate that screen behaviors are heritable, highly polygenic traits, that might share genetic signals with EA and major psychiatric disorders. Furthermore, we identified the first genomic loci associated with adolescent social media use.

According to our results, three screen behaviors – television watching, gaming, and social media use – display significant nonzero *h*^*2*^_*SNP*_ (Fig. 1, Supplementary Table 3), which are generally in line with estimates for other behavioural traits^51,52^. Furthermore, our estimates fall within the same range, but have more narrow standard errors than *h*^*2*^_*SNP*_ reported for time spent on gaming, video watching and total screen time among children^7^. To our knowledge, *h*^*2*^_*SNP*_ estimates of social media use have not been reported before.

Our study provides new perspectives on the potential shared genetic basis between screen time use and mental-health related phenotypes. We demonstrate that each of the screen behaviors displayed significant genetic correlations with one or more major psychiatric disorders (Fig. 4). The most compelling pattern of associations was observed for ADHD, which has positive genetic correlations with all three screen-based phenotypes. Although many studies have linked ADHD and problematic screen usage on a phenotypic level^53,54^, genetic studies on the topic are scarce. Overall, our findings are consistent with previous results indicating that higher genetic liability for ADHD can contribute to longer screen time utilization, and that phenotypic association between screen use and attention problems is partially explained by genetic factors^7,20,55,56^. Notably, social media use displayed positive genetic correlation with both MDD and AUD, in addition to ADHD, but was negatively correlated with ASD.

Another pattern emerged in the correlations between screen behaviours and addiction-related phenotypes. Social media use showed positive genetic correlations with both AUD and CUD, which may cautiously suggest a shared genetic liability reflecting a broader addiction-related risk pathway. In contrast, despite the recognition of “gaming disorder” as a behavioural addiction and its co-occurrence with other addictive behaviours^57^, gaming in our sample was not genetically correlated with either AUD or CUD. As this may be due to limited statistical power, future studies with larger samples will be necessary to clarify these relationships. Notably, AN showed negative genetic correlation with both gaming and social media use, despite phenotypic studies reporting associations between higher screen engagement and increased symptoms of disordered eating^58^. This discrepancy may reflect differences between clinical manifestation of illness with functional impairment and subclinical symptomatology^59^, and warrants further investigation in future studies. Overall, though our findings should be considered preliminary, they provide early indications of mechanisms by which genetic factors may increase susceptibility to both mental illness and screen behaviours, which remain to be uncovered.

Another distinct pattern was observed for EA, which displayed highly significant negative genetic correlations with television watching, gaming, and social media use. The relationship between decline in academic performance and increased screen time is well documented^15,60^, but there are no studies investigating the potentially shared genetic background underlying this association. Our results might suggest that adolescents with a high load of common genetic variants predisposing to problematic screen use may also be at higher risk for lower EA. Association between screen use and EA may at least partly be mediated by attention difficulties, which is consistent with the significant genetic correlations observed for both EA and screen behaviors with ADHD^55,61^. Indeed, re-estimating the genetic correlations between screen behaviors and ADHD while conditioning on EA revealed that most of these associations were substantially attenuated. This was also observed for other psychiatric disorders. These findings carefully suggest that the observed genetic overlap between psychiatric disorders and screen behaviours may be partially accounted for by shared genetic influences with educational attainment or broader cognitive functioning. More generally, the results cautiously point to a complex pattern of shared genetic influences linking screen use, psychiatric disorders, and EA—indicating that these traits may be interconnected through underlying genetic liability related to cognitive functioning and self-regulation. Further studies are needed to investigate this pattern in greater detail. Our sensitivity analysis indicate that the identified associations were not driven by participants with a history of mental illness (Supplementary Fig. 4). Based on the current findings, we carefully suggest that individuals with a high load of genetic risk factors for a particular psychiatric disorder (but not necessarily with the diagnosis itself) may be at higher risk for displaying more extreme screen behaviors.

By combining GWAS summary statistics on social media use and EA^27^ in the condFDR analysis^31,32^, we enhanced discovery in the moderately powered GWAS, and identified three genomic loci associated with social media use (Fig. 2, Supplementary Table 8). A more detailed discussion of the identified genomic loci is provided in the Supplementary Note, as these findings remain exploratory, though they might offer intriguing leads. More studies are warranted to further evaluate the variants identified in this study, and to clarify their biological effects.

Our study is not without limitations. Generally, selection bias is a major challenge in cohort studies, and MoBa participants were found to not be representative of the entire Norwegian population^62^. The relatively narrow age range of our sample (14–16 years) also limits generalizability, as screen use patterns may vary across the wider span of adolescence. Moreover, we were unable to estimate potential discrepancies between self-reported and objectively measured screen time, and we did not have information about the media content participants were engaging with. The complex associations between phenotypes prevents us from translating the observed genetic correlations into actual pleiotropy. The initial set of single-trait GWASs performed in our study did not unambiguously identify any loci associated with screen behaviors, though many variants reached the suggestive threshold. We hold the view that this pattern of results is merely due to low power, despite the sample being substantially larger than any prior study of screen behaviors. Insufficient statistical power prevents us from identifying causal relationships between screen behaviors and mental disorders – MR results were inconclusive even for the most robust GWAS dataset. Therefore, we urge the research community to continue collecting large-scale data about screen-based activities, as it will greatly improve our understanding of one of the most widespread modern behavioural phenotypes.

In addition, we note that the analyses in the present study were restricted to individuals of European ancestry. This represents a clear limitation, as it reduces the generalisability of our findings – for example, heritability and correlation estimates may not translate directly to other populations – and reflects a broader, though increasingly recognised imbalance in genomic research. There is a need for greater ancestral diversity in future genetic studies of screen behaviours, which, despite their global relevance, remain largely understudied in non-European populations.

In summary, we demonstrated that television watching, gaming, and social media use are heritable, highly polygenic traits, which display significant genetic correlations with one or more major psychiatric disorders, and are negatively correlated with EA. Furthermore, we identified three genomic loci associated with adolescent social media use. Though our results should be interpreted with caution given the rather low statistical power of the current study, they offer early insights into the genetics of screen behaviors and may generate new hypotheses regarding the relationship between screen time use, mental health, and EA during adolescence.

## Supplementary Information

Supplementary Information 1.

Supplementary Information 2.

## Data Availability

The datasets supporting the conclusions of this article are available from the Norwegian Institute of Public Health, but restrictions apply to the availability of these data. The study website provides details on how to access data and information on the available variables (https://www.fhi.no/en/ch/studies/moba/for-forskere-artikler/research-and-data-access/). The summary statistics from GWAS conducted in this study will be made publicly available on the Norwegian Institute of Public Health website (https://www.fhi.no/en/ch/studies/moba/for-forskere-artikler/gwas-data-from-moba/).
