# Supplementary material for: Genome-wide analysis of screen behaviors among adolescents identifies novel loci and overlap with educational attainment and mental disorders
Source: Sci Rep. 2025 Oct 2;15:34420. doi: 10.1038/s41598-025-17450-y (PMC12491421; doi:10.1038/s41598-025-17450-y)
Supplement: Supplementary file 1 — Supplementary Information 1. [file 41598_2025_17450_MOESM1_ESM.docx]

**Supplementary Note**

[Supplementary Methods 1](#_Toc202444862)

[Study sample 1](#_Toc202444863)

[Genome-Wide Association Analyses 2](#_Toc202444864)

[Conditional False Discovery Rate (condFDR) analyses 2](#_Toc202444865)

[Functional Analyses 2](#_Toc202444866)

[Estimation of SNP-Based Heritabilities and Genetic Correlations 3](#_Toc202444867)

[Mendelian Randomization 3](#_Toc202444868)

[Genomic Structural Equation Modelling 3](#_Toc202444869)

[Supplementary Discussion 4](#_Toc202444870)

[Supplementary Figures 6](#_Toc202444871)

[References 10](#_Toc202444872)

# Supplementary Methods

## Study sample

Biological samples were sent to the Norwegian Institute of Public Health where DNA was extracted by standard methods and stored. Genotyping of the MoBa cohort was conducted through multiple research projects spanning several years, involving various selection criteria, and genotyping centres. Full details about the genotyping and quality control procedures are provided elsewhere ^1^. Additional information on how to access the MoBaPsychGen post-imputation QC data is available here: https://www.fhi.no/en/more/research-centres/psychgen/access-to-genetic-data-after-qualitycontrol-by-the-mobapsychgen-pipeline-v/.

Questionnaire data used in the current study were collected between 2017-2023. Mothers participating in MoBa gave written informed consent on behalf of their children. At 18, the children will be asked to give renewed consent for the continued use of data collected while they were younger. At 14, the adolescents were informed about the study and asked to provide new consent for participation in follow-up investigations. Data of participants who withdrew their consent before August 2024 are not included in the analyses.

Data about psychiatric diagnoses were retrieved from the Norwegian Patient Registry (NPR), which contains ICD-10 coded diagnoses registered in specialist healthcare services.

## Genome-Wide Association Analyses

To ensure that screen use patterns reflect the general adolescent population as accurately as possible, the sample was restricted to participants without a high likelihood of severe disability. Therefore, in line with the Adolescent Brain Cognitive Development Study criteria ^2^, we excluded individuals with current diagnosis of schizophrenia (F20-F29), mental impairment/intellectual disability (F70-F79), alcohol/substance use disorder (F10-F19), and autism spectrum disorder (F84), based on the data from the NPR. Moreover, subjects with ambiguous sex and subjects with chromosomal abnormalities were removed from analyses. This resulted in a sample of 17,945 individuals. For each pair of participants with a kinship coefficient greater than 0.05, one member was randomly excluded from analyses, resulting in a sample of 16,027 unrelated individuals.

GWASs were conducted using an additive multivariate linear regression model with PLINK2 ^3^. All summary statistics underwent quality control and were cleaned using the cleansumstats pipeline ^4^. Analyses were conducted using singularity containers ^5^.

## Conditional False Discovery Rate (condFDR) analyses

The condFDR approach is described in detail elsewhere^6,7^. Briefly, it boosts GWAS discovery by leveraging overlapping SNP associations between two GWASs to re-rank the test statistics in a primary phenotype conditional on the associations in a secondary phenotype.

To visualize cross-trait enrichment we used conditional quantile-quantile (QQ) plots, which show *p*-value distributions for a primary trait for all SNPs, and for SNP strata set by their association with a secondary trait. For QQ plots production, we excluded variants within regions with complex linkage disequilibrium (LD) structure (MHC region: chr6:25119106–33854733, and 8p23 inversion: chr8:7200000–12500000, GRCh37 coordinates). Successive leftward deflection of the variant strata with increasing significance in the conditional phenotype in both directions suggests strong cross-trait enrichment.

## Functional Analyses

Genomic loci were defined by identifying independent significant SNPs with condFDR < 0.01 that were not in close LD with each other (r^2^ < 0.60), according to the FUMA protocol^8^. Lead SNPs were then defined by the independent significant SNPs with r^2^ < 0.1 in approximate LD. Candidate SNPs were defined as all SNPs with condFDR < 0.10 and in LD (r^2^ ≥ 0.60) with an independent significant SNP. The loci borders were set by identifying all candidate SNPs in LD (r^2^ ≥ 0.6) with one of the independent significant SNPs in the locus. Loci < 250 kb apart were merged, and the lead SNP of the merged locus was selected as the SNP with the most significant condFDR value. Overlapping signals within complex LD regions were represented by one independent lead SNP only. All LD r^2^ values were obtained from the 1000 Genomes Project European-ancestry haplotype reference panel^9^.

## Estimation of SNP-Based Heritabilities and Genetic Correlations

We used the set of LD scores provided by the software’s creators, based on the 1000 Genomes Project’s European sample. Summary statistics from external GWASs of schizophrenia, bipolar disorder, major depressive disorder, autism spectrum disorder, alcohol use disorder, and educational attainment were harmonized using the cleansumstats pipeline. Additional SNP quality control routines were equivalent to the defaults employed with the LDSC *munge_sumstats.py* function. Following the recommended practices, we assumed no sample overlap. Analyses were done using Python ver. 3.9.5.

## Mendelian Randomization

Mendelian Randomization (MR) rely on three main instrumental variable assumptions: relevance, independence, and exclusion restriction. MR analyses were performed using the R package TwoSampleMR^10^. We applied several MR methods: inverse variance weighted^11^, weighted median^12^, and MR Egger^13^. Genetic instruments for ADHD were selected at a *p*-value threshold of 5×10^-8^. Genetic instruments for social media use were selected at a relaxed *p*-value threshold of 1×10^-5^ to account for the lower power of the GWAS, in line with previous studies’ that adopted more relaxed *p*-value thresholds for underpowered GWASs^14,15^. Analyses were done using R ver. 4.4.1.

# Genomic Structural Equation Modelling

We used genomic structural equation modelling (SEM)^16^ to estimate how the genetic correlations between screen-based behaviours and psychiatric disorders change when conditioning on educational attainment (EA). Specifically, EA was modelled as a covariate in the genetic covariance structure (e.g., ‘ADHD ~ EA, SoMe ~ EA, ADHD ~~ SoMe’). The analysis was restricted to psychiatric phenotypes that showed significant genetic correlations with both EA and at least one screen-based behaviour. The modelling was performed in the GenomicSEM R package v0.0.5 (https://github.com/GenomicSEM/GenomicSEM).

# Supplementary Discussion

Here we aim to discuss the identified genomic loci associated with social media use in more detail, while acknowledging that these interpretations remain largely exploratory yet intriguing.

The lead SNPs were mapped to putative causal genes using the Variant to Gene (V2G) tool from the open-source OpenTargets Genetics^17–19^. This platform was also used to inspect associations of the mapped genes with other phenotypes. To estimate expression of the mapped genes in human brain, we used the open-source Brain RNA-Seq Database^20^.

The most significant variant (rs7110805, intergenic) is located on chromosome 11 and represents a broad region of associations, which contains three protein-coding genes: *MTMR2, FAM67B,* and *CEP57.* *MTMR2* encodes myotubularin‑related protein 2 and is moderately expressed in the brain^21^, mostly in oligodendrocytes and neurons. Its neural functions remain unclear, though synaptically localized *MTMR2* might maintain excitatory synapses by inhibiting excessive endosomal production and destructive trafficking to lysosomes^22^. According to the Open Targets Genetics model^18,19^, *MTMR2* is also predicted to be likely casual (L2G pipeline scores > 0.6) for EA^23,24^, consistent with our hypothesis about shared genetic factors between social media use and EA. *FAM67B* gene encodes its eponymous protein, with low brain expression levels. Nevertheless, it has a moderate chance to be casual at locus discovered in the GWAS of vertex-wise sulcal depth (L2G score = 0.54)^25^. Finally, the *CEP57* gene, which encodes centrosomal protein 57, is moderately expressed in neurons and fetal astrocytes^21^. The CEP57 protein controls centriole duplication and centrosome maturation for faithful cell division. Interestingly, *CEP57* has more than a 50% chance to be casual at loci discovered in GWAS for leisure television watching, cortical surface area, and cognitive aspects of EA^26–28^. Again, these findings are consistent with our hypothesis of shared genetic basis between EA and screen behaviors.

The second locus associated with social media use is represented by an intergenic variant rs359240 on chromosome 2, with no protein-coding genes in the direct vicinity (Figure 3A). Nevertheless, this variant has a CADD score of 19.6, suggesting high deleteriousness^29^. Interestingly, rs359240 crossed genome-wide suggestive significance threshold for several brain-related and behavioral traits, like smoking, risk-taking behavior, and age at first sexual intercourse and age at first live birth^30–32^.

Finally, the third identified locus was located on chromosome 4, and is represented by an ncRNC intronic variant rs6848288, which has multiple LD-linked variants with low condFDR values (Figure 3B). The OpenTargets platform links rs6848288 most strongly to the *SMARCAD1* gene*,* which encodes its eponymous protein and participates in transcriptional regulation, heterochromatin maintenance, DNA repair, and replication, though the molecular basis of its role in these processes is not fully understood^33^. The *SMARCAD1* gene is weakly expressed in both human astrocytes and neurons^21^. However, according to the L2G pipeline, this gene has more than a 70% chance to be causal at loci discovered in GWAS for cortical thickness and vertex-wise surface area^25,28^. The link between putative causal genes for screen behaviors and brain structures measures is intriguing, especially in light of studies that identified associations between internet gaming disorder and structural and functional brain changes ^34^. Brain changes associated to a more moderate level of digital media use remain to be investigated, and further studies in adolescent population are warranted.

Functional characterization of the identified loci by FUMA revealed two exonic nonsynymous SNPs located on chromosome 11 (Supplementary Table 9). However, like in GWAS on other complex human traits, most of the identified candidate SNPs reside in non-coding DNA, suggesting a regulatory and more indirect effects^35^. This may also indicate that the loci do not influence a distinct biological process but represent non-specific genetic effects common to several mental health related phenotypes. These findings may also reflect insufficient statistical power. Nevertheless, follow-up studies are warranted to determine the specific causal genetic variants underlying the detected loci.

Results of the Mendelian Randomization (MR) analysis did not reveal any significant causal relationships. A weak trend suggested that genetic liability to ADHD may be associated with increased social media use (p < 0.1); however, given the limited statistical power combined with the methodological challenges of applying MR to highly polygenic traits, these findings should be interpreted with caution and considered inconclusive.

Supplementary Figures
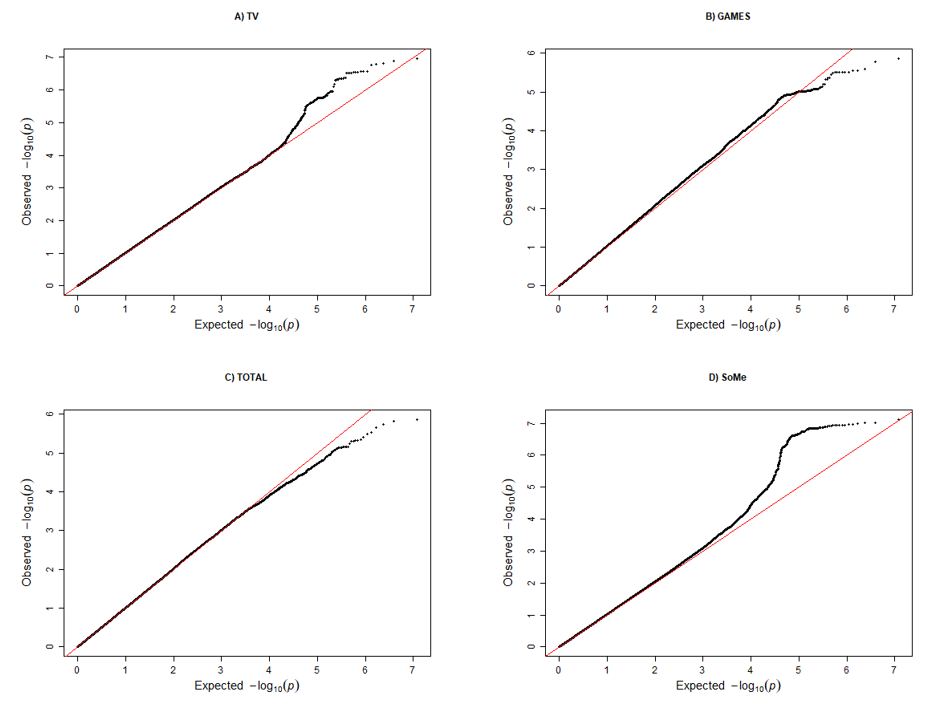


**Supplementary Figure 1.** Quantile-Quantile plots of p-values from genome-wide association studies of the screen behaviors in the MoBa cohort (n = 16 027).

TV: watching movies/series/TV; GAMES: playing games on PC, TV, tablet, mobile, etc.; TOTAL: sitting/lying with PC, mobile, or tablet; SoMe: communicating with friends on social media.

**
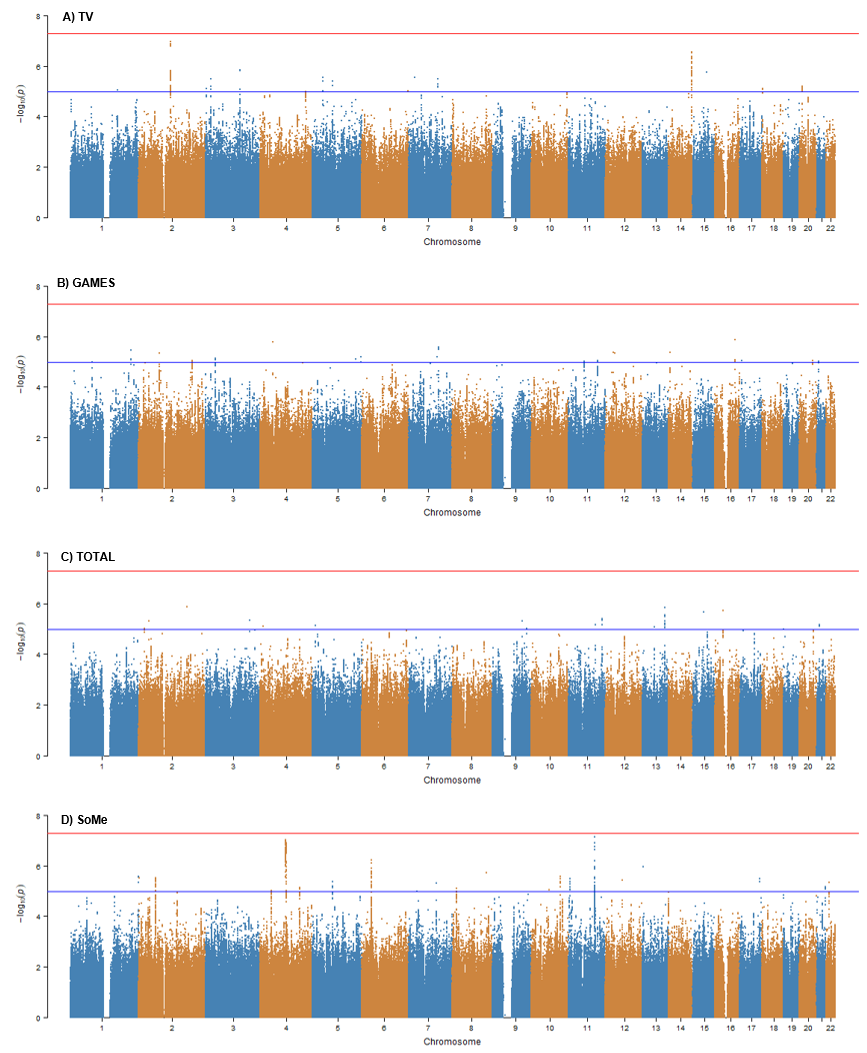
**

**Supplementary Figure 2.** Manhattan plots of genome-wide association studies of the screen behaviors in the MoBa cohort (n = 16 027).

The x-axis shows genomic position (chromosomes 1–22), and the y-axis shows statistical significance as –log10(p-value). The blue line indicates the genome-wide suggestive threshold (p < 1 × 10^−5^), the red line indicates the genome-wide significance threshold (p < 5 × 10^−8^).

TV: watching movies/series/TV; GAMES: playing games on PC, TV, tablet, mobile, etc.; TOTAL: sitting/lying with PC, mobile, or tablet; SoMe: communicating with friends on social media.


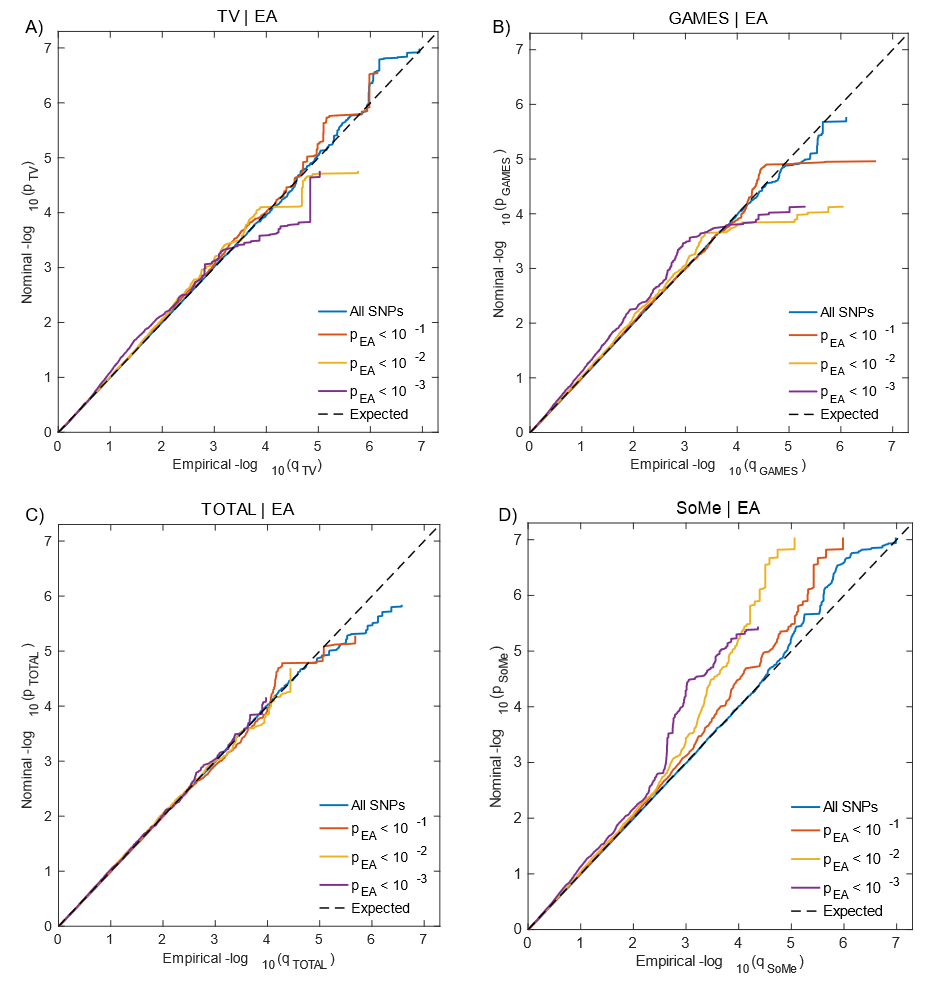


**Supplementary Figure 3.** Conditional Quantile-Quantile (QQ) plots.

QQ plots demonstrate relation between expected (x-axis) and observed (y-axis) significance of markers in the primary trait when markers are stratified by their p-values in the conditional trait.

Screen time use phenotypes from the MoBa cohort are conditioned on the educational attainment (EA) phenotype. A sequence of four nested strata is presented: all single nucleotide polymorphisms (SNPs) (i.e. p-values of the secondary trait ≤ 1.00) (blue), pconditional_trait < 0.1 (red), pconditional_trait < 0.01 (yellow), and pconditional_trait < 0.001 (purple). Dashed black line demonstrates expected behaviour under no association. Increasing degree of leftward deflection from the no‑association line for strata of SNPs with higher significance in the conditional trait indicates polygenic overlap.

TV: watching movies/series/TV; GAMES: playing games on PC, TV, tablet, mobile, etc.; TOTAL: sitting/lying with PC, mobile, or tablet; SoMe: communicating with friends on social media.


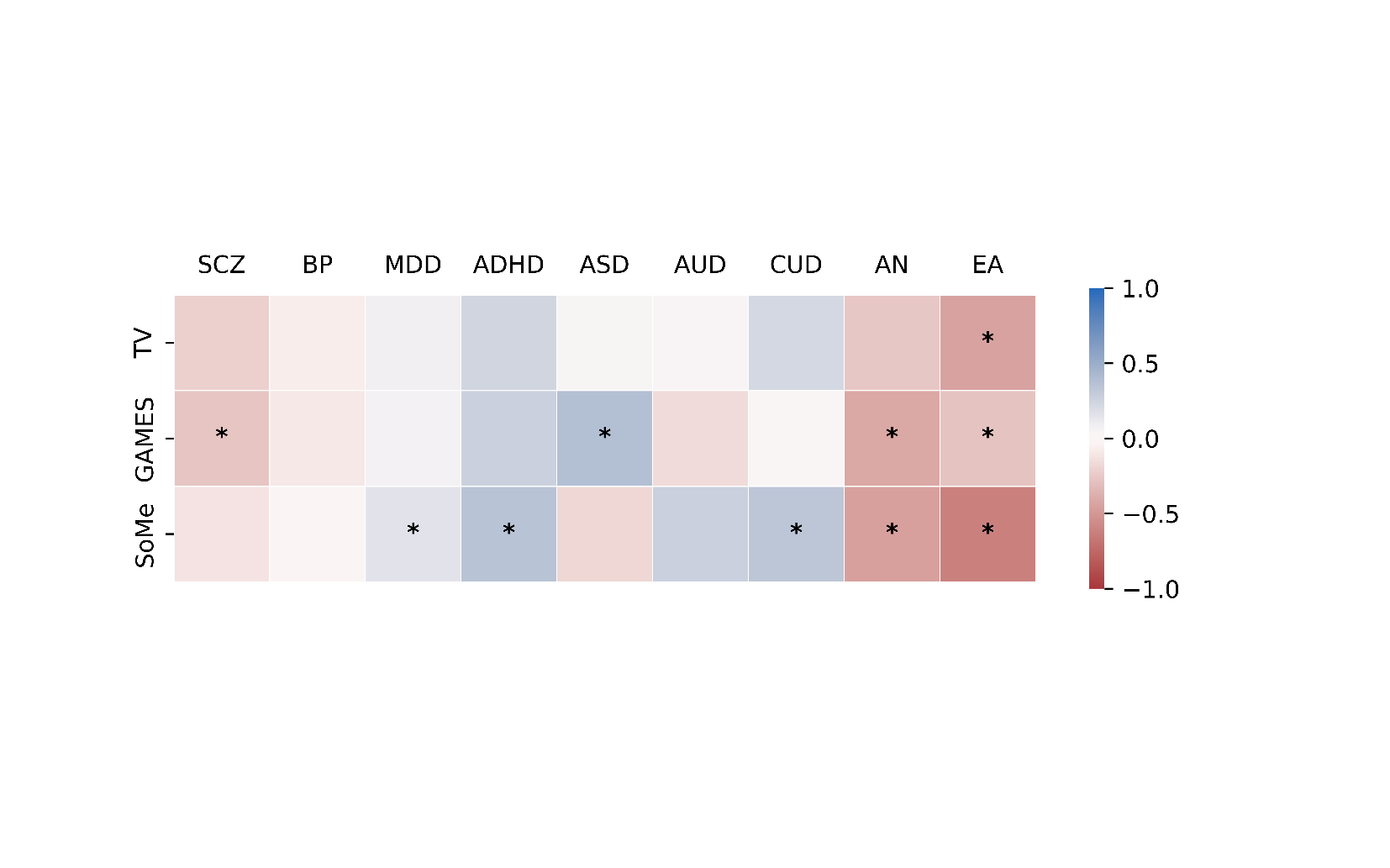


**Supplementary Figure 4.** Genetic correlation estimates between screen-based behaviors and eight major psychiatric disorders and educational attainment, subsample of participants without a history of any psychiatric disorder (n = 13 375).

Asterisks indicate significant estimates at FDR < 0.05 (Benjamini-Hochberg procedure).

TV: watching movies/series/TV; GAMES: playing games on PC, TV, tablet, mobile, etc.; SoMe: communicating with friends on social media; SCZ, schizophrenia; BP, bipolar disorder; MDD, major depressive disorder; ASD, autism spectrum disorder; ADHD, attention deficit hyperactivity disorder; AUD, alcohol use disorder; CUD, cannabis use disorder; AN, anorexia nervosa; EA, educational attainment.

# References

1. Corfield, E. C., Shadrin, A. A., Frei, O., Rahman, Z., Lin, A., Athanasiu, L., *et al.* The Norwegian Mother, Father, and Child cohort study (MoBa) genotyping data resource: MoBaPsychGen pipeline v.1. *bioRxiv* (2024) doi:10.1101/2022.06.23.496289.

2. ABCD Study. https://abcdstudy.org/.

3. Purcell, S., Neale, B., Todd-Brown, K., Thomas, L., Ferreira, M. A. R., Bender, D., *et al.* PLINK: A tool set for whole-genome association and population-based linkage analyses. *Am. J. Hum. Genet.* **81**, 559–575 (2007).

4. Gadin, J. R., Zetterberg, R., Meijsen, J. & Schork, A. J. Cleansumstats: Converting GWAS sumstats to a common format to facilitate downstream applications. *Zenodo* https://github.com/BioPsyk/cleansumstats (2023).

5. Akdeniz, B. C., Frei, O., Hagen, E., Filiz, T. T., Karthikeyan, S., Pasman, J., *et al.* COGEDAP: A COmprehensive GEnomic Data Analysis Platform. *arXiv: 2212.14103* (2022).

6. Andreassen, O. A., Thompson, W. K., Schork, A. J., Ripke, S., Mattingsdal, M., Kelsoe, J. R., *et al.* Improved Detection of Common Variants Associated with Schizophrenia and Bipolar Disorder Using Pleiotropy-Informed Conditional False Discovery Rate. *PLoS Genet.* **9**, e1003455 (2013).

7. Smeland, O. B., Frei, O., Shadrin, A., O’Connell, K., Fan, C. C., Bahrami, S., *et al.* Discovery of shared genomic loci using the conditional false discovery rate approach. *Human Genetics* vol. 139 85–94 at https://doi.org/10.1007/s00439-019-02060-2 (2020).

8. Watanabe, K., Taskesen, E., Van Bochoven, A. & Posthuma, D. Functional mapping and annotation of genetic associations with FUMA. *Nat. Commun.* **8**, 1–11 (2017).

9. Auton, A., Abecasis, G. R., Altshuler, D. M., Durbin, R. M., Bentley, D. R., Chakravarti, A., *et al.* A global reference for human genetic variation. *Nature* **526**, 68–74 (2015).

10. Hemani, G., Zheng, J., Elsworth, B., Wade, K. H., Haberland, V., Baird, D., *et al.* The MR-base platform supports systematic causal inference across the human phenome. *Elife* **7**, (2018).

11. Burgess, S., Butterworth, A. & Thompson, S. G. Mendelian randomization analysis with multiple genetic variants using summarized data. *Genet. Epidemiol.* **37**, 658–665 (2013).

12. Bowden, J., Davey Smith, G., Haycock, P. C. & Burgess, S. Consistent Estimation in Mendelian Randomization with Some Invalid Instruments Using a Weighted Median Estimator. *Genet. Epidemiol.* **40**, 304–314 (2016).

13. Bowden, J., Smith, G. D. & Burgess, S. Mendelian randomization with invalid instruments: Effect estimation and bias detection through Egger regression. *Int. J. Epidemiol.* **44**, 512–525 (2015).

14. Li, F., Tang, M., Hao, C., Yang, M., Pan, Y. & Lei, P. Brain imaging traits and epilepsy: Unraveling causal links via mendelian randomization. *Brain Behav.* **14**, e70051 (2024).

15. Sanna, S., van Zuydam, N. R., Mahajan, A., Kurilshikov, A., Vich Vila, A., Võsa, U., *et al.* Causal relationships among the gut microbiome, short-chain fatty acids and metabolic diseases. *Nature Genetics* vol. 51 600–605 at https://doi.org/10.1038/s41588-019-0350-x (2019).

16. Grotzinger, A. D., Rhemtulla, M., de Vlaming, R., Ritchie, S. J., Mallard, T. T., Hill, W. D., *et al.* Genomic structural equation modelling provides insights into the multivariate genetic architecture of complex traits. *Nat. Hum. Behav.* **3**, 513–525 (2019).

17. Ghoussaini, M., Mountjoy, E., Carmona, M., Peat, G., Schmidt, E. M., Hercules, A., *et al.* Open Targets Genetics: Systematic identification of trait-associated genes using large-scale genetics and functional genomics. *Nucleic Acids Res.* **49**, D1311–D1320 (2021).

18. Ochoa, D., Hercules, A., Carmona, M., Suveges, D., Baker, J., Malangone, C., *et al.* The next-generation Open Targets Platform: reimagined, redesigned, rebuilt. *Nucleic Acids Res.* **51**, D1353–D1359 (2023).

19. Mountjoy, E., Schmidt, E. M., Carmona, M., Schwartzentruber, J., Peat, G., Miranda, A., *et al.* An open approach to systematically prioritize causal variants and genes at all published human GWAS trait-associated loci. *Nat. Genet.* **53**, 1527–1533 (2021).

20. Brain RNA-Seq. https://brainrnaseq.org/.

21. Zhang, Y., Sloan, S. A., Clarke, L. E., Caneda, C., Plaza, C. A., Blumenthal, P. D., *et al.* Purification and Characterization of Progenitor and Mature Human Astrocytes Reveals Transcriptional and Functional Differences with Mouse. *Neuron* **89**, 37–53 (2016).

22. Lee, H. W., Kim, Y., Han, K., Kim, H. & Kim, E. The phosphoinositide 3-phosphatase MTMR2 interacts with PSD-95 and maintains excitatory synapses by modulating endosomal traffic. *J. Neurosci.* **30**, 5508–5518 (2010).

23. Kichaev, G., Bhatia, G., Loh, P. R., Gazal, S., Burch, K., Freund, M. K., *et al.* Leveraging Polygenic Functional Enrichment to Improve GWAS Power. *Am. J. Hum. Genet.* **104**, 65–75 (2019).

24. Lee, J. J., Wedow, R., Okbay, A., Kong, E., Maghzian, O., Zacher, M., *et al.* Gene discovery and polygenic prediction from a genome-wide association study of educational attainment in 1.1 million individuals. *Nat. Genet.* **50**, 1112–1121 (2018).

25. Van Der Meer, D., Kaufmann, T., Shadrin, A. A., Makowski, C., Frei, O., Roelfs, D., *et al.* The genetic architecture of human cortical folding. *Sci. Adv.* **7**, eabj9446–eabj9446 (2021).

26. van de Vegte, Y. J., Said, M. A., Rienstra, M., van der Harst, P. & Verweij, N. Genome-wide association studies and Mendelian randomization analyses for leisure sedentary behaviours. *Nat. Commun.* **11**, 1–10 (2020).

27. Demange, P. A., Malanchini, M., Mallard, T. T., Biroli, P., Cox, S. R., Grotzinger, A. D., *et al.* Investigating the genetic architecture of noncognitive skills using GWAS-by-subtraction. *Nat. Genet.* **53**, 35–44 (2021).

28. Shadrin, A. A., Kaufmann, T., van der Meer, D., Palmer, C. E., Makowski, C., Loughnan, R., *et al.* Vertex-wise multivariate genome-wide association study identifies 780 unique genetic loci associated with cortical morphology. *Neuroimage* **244**, 118603–118603 (2021).

29. Kircher, M., Witten, D. M., Jain, P., O’roak, B. J., Cooper, G. M. & Shendure, J. A general framework for estimating the relative pathogenicity of human genetic variants. *Nat. Genet.* **46**, 310–315 (2014).

30. Clifton, E. A. D., Perry, J. R. B., Imamura, F., Lotta, L. A., Brage, S., Forouhi, N. G., *et al.* Genome–wide association study for risk taking propensity indicates shared pathways with body mass index. *Commun. Biol.* **1**, 1–10 (2018).

31. Mills, M. C., Tropf, F. C., Brazel, D. M., van Zuydam, N., Vaez, A., Agbessi, M., *et al.* Identification of 371 genetic variants for age at first sex and birth linked to externalising behaviour. *Nat. Hum. Behav.* **5**, 1717–1730 (2021).

32. Pan UKBB Team. Pan UKBB. https://pan.ukbb.broadinstitute.org/.

33. Sachs, P., Bergmaier, P., Treutwein, K. & Mermoud, J. E. The Conserved Chromatin Remodeler SMARCAD1 Interacts with TFIIIC and Architectural Proteins in Human and Mouse. *Genes (Basel).* **14**, 1793 (2023).

34. Jeong, B. S., Han, D. H., Kim, S. M., Lee, S. W. & Renshaw, P. F. White matter connectivity and Internet gaming disorder. *Addict. Biol.* **21**, 732–742 (2016).

35. Sullivan, P. F. & Geschwind, D. H. Defining the Genetic, Genomic, Cellular, and Diagnostic Architectures of Psychiatric Disorders. *Cell* vol. 177 162–183 at https://doi.org/10.1016/j.cell.2019.01.015 (2019).
